# Hemoglobin Concentration and Pregnancy Outcomes: A Systematic Review and Meta-Analysis

**DOI:** 10.1155/2013/769057

**Published:** 2013-07-25

**Authors:** Bunyarit Sukrat, Chumpon Wilasrusmee, Boonying Siribumrungwong, Mark McEvoy, Chusak Okascharoen, John Attia, Ammarin Thakkinstian

**Affiliations:** ^1^Section for Clinical Epidemiology and Biostatistics, Faculty of Medicine, Ramathibodi Hospital, Mahidol University, Rama VI Road, Rachatevi, Bangkok 10400, Thailand; ^2^Bureau of Reproductive Health, Department of Health, Ministry of Public Health, Muang, Nontaburi 11000, Thailand; ^3^Department of Surgery, Faculty of Medicine, Ramathibodi Hospital, Mahidol University, Rama VI Road, Rachatevi, Bangkok 10400, Thailand; ^4^Department of Surgery, Faculty of Medicine, Thammasat Hospital, Thammasat University, Klongluang, Pathumthani 12121, Thailand; ^5^Centre for Clinical Epidemiology and Biostatistics, The University of Newcastle, Newcastle, NSW 2300, Australia; ^6^Department of Pediatrics, Faculty of Medicine, Ramathibodi Hospital, Mahidol University, Rama VI Road, Rachatevi, Bangkok 10400, Thailand

## Abstract

*Objective*. To conduct a systematic review and meta-analysis of hemoglobin effect on the pregnancy outcomes. *Methods*. We searched MEDLINE and SCOPUS from January 1, 1990 to April 10, 2011. Observational studies addressing association between hemoglobin and adverse pregnancy outcomes were selected. Two reviewers independently extracted data. A mixed logistic regression was applied to assess the effects of hemoglobin on preterm birth, low birth weight, and small for gestational age. *Results*. Seventeen studies were included in poolings. Hemoglobin below 11 g/dL was, respectively, 1.10 (95% CI: 1.02–1.19), 1.17 (95% CI: 1.03–1.32), and 1.14 (95% CI: 1.05–1.24) times higher risk of preterm birth, low birth weight, and small for gestational age than normal hemoglobin in the first trimester. In the third trimester, hemoglobin below 11 g/dL was 1.30 (95% CI: 1.08–1.58) times higher risk of low birth weight. Hemoglobin above 14 g/dL in third trimester decreased the risk of preterm term with ORs of 0.50 (95% CI: 0.26–0.97), but it might be affected by publication bias. *Conclusions*. Our review suggests that hemoglobin below 11 g/dl increases the risk of preterm birth, low birth weight, and small gestational age in the first trimester and the risk of low birth weight in the third trimester.

## 1. Introduction

Anemia has been claimed to be the most common nutritional disorder in pregnancy across the world [1, 2]. The worldwide prevalence is estimated at 41.8% (95% CI: 39.9–43.8) [2] and is more common in African (57.1%, 95% CI: 52.8–61.3) pregnant women. The prevalence, however, depends on the definition of anemia, in which two definitions are commonly used, that is, the Centers for Disease Control and Prevention (CDC) and the World Health Organization (WHO) definitions [3, 4]. Adverse pregnancy outcomes thought to be affected by anemia include maternal mortality, perinatal mortality, preterm birth (PTB), low birth weight (LBW), and small for gestational age (SGA). Previous research has demonstrated a strong association between severe anemia and maternal mortality [5], but the risk of maternal mortality in pregnant women with moderate anemia (i.e., hemoglobin concentration of 40–80 g/dL) was inconclusive. The impact of anemia on other adverse pregnancy outcomes (e.g., PTB, LBW and SGA) is controversial. Some studies found significant associations [6–12], while other studies did not [13–16]; this is likely the result of different studies using different criteria or cutoff thresholds for defining anemia. In another way, some studies reported the association between high hemoglobin concentration and adverse pregnancy outcomes [17, 18]. Although a previous systematic review published in 2000 [19] reported maternal anemia in early pregnancy (<20 gestational week) increased the risk of PTB but not for LBW and SGA. Most of the included studies in this review were from developed countries, except 1 study was from African [20], and 2 studies were from China [9, 21]. Anemic pregnancy was more prevalent in developing countries; thus the effects of anemia on pregnancy outcomes in developing countries were still in question. We therefore conducted a systematic review to determine the association of hemoglobin concentration and adverse pregnancy outcomes including PTB, LBW, and SGA in each trimester of pregnancy.

## 2. Material and Methods

### 2.1. Identification of Studies

Studies were identified from MEDLINE and SCOPUS from January 1, 1990 to April 10, 2011. Reference lists from selected articles, narrative reviews, and systematic reviews were also reviewed to find relevant articles that were not identified by the initial search strategies. The following search terms were used: pregnancy, pregnant women, hemoglobin/haemoglobin, anemia/anaemia, hematologic/haematologic parameter, mortality, preterm birth/delivery, low birth weight, and small for gestational age. Search strategies are clearly described in Appendix 1  in the Supplementary Material available online at http://dx.doi.org/10.1155/2013/769057. Only human studies published in English were considered.

### 2.2. Study Selection

Eligibility assessment was performed by one reviewer using the following inclusion criteria. Any observational study (i.e., case control or cohort) performed in singleton pregnancy that assessed the association between hemoglobin concentration and any adverse pregnancy outcomes (i.e., still births, neonatal mortality, perinatal mortality, LBW, PTB, and SGA) reported gestational age at the time of hemoglobin testing and had sufficient data to allow calculation of the odds ratio and 95% confidence interval for dichotomous outcomes, number of subjects, mean and standard deviation according to hemoglobin concentration groups for continuous outcomes.

### 2.3. Data Extraction

 The data extraction was performed independently by two reviewers. Data on study characteristics (i.e., maternal age, ethnicity, gestational age at first antenatal visit, gestational age at delivery, parity, number of antenatal care visits, and smoking), mean and standard deviation of continuous outcomes, and frequencies of crosstabulation between each hemoglobin group and outcome for categorical data were extracted. Any disagreement was resolved by consensus between the two reviewers. If no agreement could be reached, it was adjudicated by a third reviewer.

### 2.4. Risk of Bias Assessment

 A risk of bias assessment was independently performed by 2 reviewers. The tool use was modified from meta-analysis for genetic association studies using epidemiological part [22]. Four domains were assessed, which were representativeness of subjects, ascertainment of outcomes, ascertainment of exposures, and confounding bias. Disagreements between the two reviewers were solved by the third author.

### 2.5. Outcomes of Interest

 The outcomes of interests were PTB, LBW, and SGA. Briefly, LBW was defined as a newborn with weight at birth of less than 2500 g. PTB was defined as a neonate born before 37 weeks gestational age (the 259th day). SGA was a newborn whose birth weight was below the 10th percentile for gestational age.

### 2.6. Statistical Analysis

 Characteristics of all included studies were described including study design, number of participants, hemoglobin cutoff, trimester, and pregnancy outcomes. Hemoglobin concentration was categorized as <9, <10, <11, 11–13.9, and ≥14 g/dL in mutually exclusive fashion for each study. To assess hemoglobin effects, data were pooled separately according to the trimester and pregnancy outcomes using the cutoff of 11–13.9 g/dL as the reference group. To compare pregnancy outcomes between multiple hemoglobin cutoffs in the same time, summary data of hemoglobin cutoffs and outcome groups were then expanded to individual patient data using the expand command in STATA. A mixed logit model with a random intercept (i.e., to account for between-study variation) was applied to assess hemoglobin effects on pregnancy outcome. The estimated pooled odds ratio (OR) along with 95% confidence interval (CI) was estimated by exponential logit coefficients. A degree of heterogeneity was estimated using multivariate meta-analysis method [23]. All analyses were performed using STATA version 12. The statistical significance was set to two-sided *P* < 0.05 for all analyses.

## 3. Results

**Figure 1:**
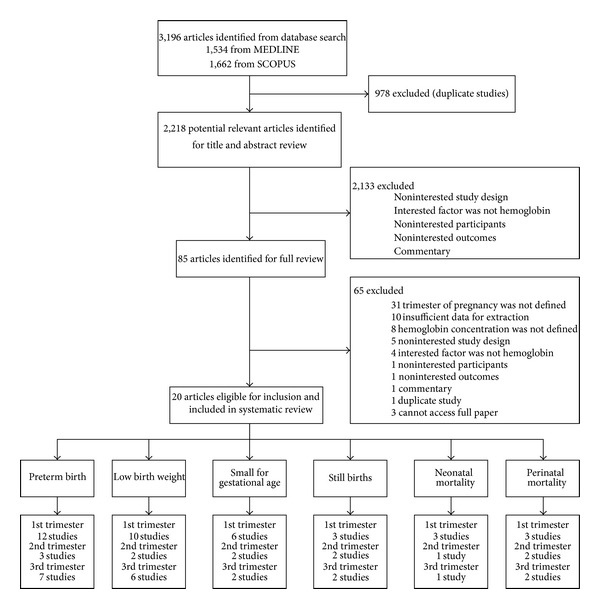
Flow of study selection.

 Eighty-five potentially relevant articles were identified, of which 65 studies were excluded, leaving 20 studies [7, 9–12, 15–18, 24–34] for data extraction. The reasons for exclusion are described in Figure 1. Characteristics of all included studies are summarized in Table 1. Briefly, 50% percent of included studies were prospective cohorts. Mean age ranged from 16.1 to 30.6 years. Most of the eligible studies included pregnant women from Asian populations (12 studies), followed by European (4 studies), Africans (2 studies), and North Americans (2 studies). PTB, LBW, and SGA were outcomes of interests in 15, 14, and 6 studies, respectively. Still births, neonatal deaths, and perinatal deaths were less frequently reported only in 5, 3, and 4 studies, respectively, and we therefore did not pool these outcomes. The number of studies available for analyses according to trimester and outcome is shown in Figure 1.

Risk of bias was performed independently by the two reviewers with the total agreement rate of 98.75% with the kappa statistic of 0.946 (*P* < 0.001). For those items where there were disagreements, the senior reviewer (AT) had performed risk of bias and made decisions. Results of assessments for individual studies were described in Supplementary Table 1. The highest quality was in the domain of ascertainment of outcome (100.0% low risk) followed by representativeness of subjects (95.0%), while the lowest was ascertainment of hemoglobin test or definition (70.0%). 

### 3.1. Preterm Birth

**Figure 2:**
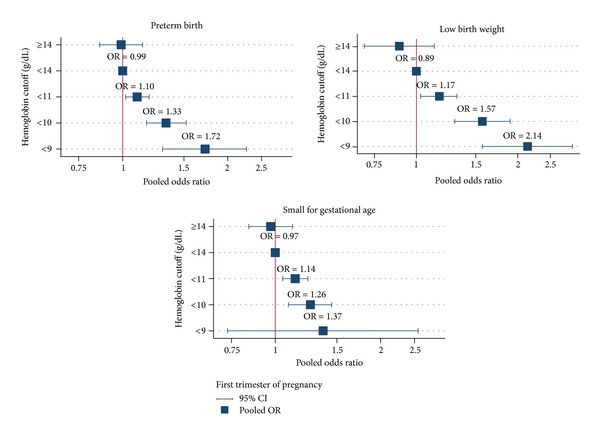
Pooling effects of hemoglobin concentration on pregnancy outcomes in the first trimester.

Eleven studies [9–12, 16–18, 27, 28, 32, 34] assessed the association between hemoglobin concentration in the first trimester and PTB. The use of hemoglobin cutoffs varied from ≤7 to ≥16 g/dL (Supplementary Table 2); we therefore recategorized the cutoffs as <9, <10, <11, 11–13.9, and ≥14 g/dL with the number of pregnant women totaling 192,870. The mixed logistic model with random intercept was applied by fitting hemoglobin cutoffs in the model using the cutoff of 11–13.9 g/dL as the reference group. The results suggested that pregnant women with hemoglobin concentration below 9, 10, and 11 g/dL had, respectively, 72% (OR: 1.72, 95% CI: 1.30–2.26), 33% (OR: 1.33, 95% CI: 1.17–1.52), and 10% (OR: 1.10, 95% CI: 1.02–1.29) higher risk of PTBs compared with pregnant women with hemoglobin concentration 11–13.9 g/dL. Hemoglobin above 14 g/dL did not increase the risk (OR: 0.99, 95% CI: 0.86–1.14); see Table 2 and Figure 2. Pooling hemoglobin effects were homogenous across studies with degrees of heterogeneity of 4% (95% CI: 0–45), 10% (95% CI: 0–59), and 5% (95% CI: 0–44) for hemoglobin cutoffs <9, <10, and <11 g/dL, respectively. For hemoglobin cutoffs >14 g/dL, the degree of heterogeneity was moderately heterogeneous with *I* ^2^ equal to 31 (95% CI: 0–74). There was no evidence of small study effect suggested by Egger tests with coefficients of 0.018 (*P* = 0.849), 0.667 (*P* = 0.745), 0.502 (*P* = 0.740), and 0.127 (*P* = 0.672), respectively.

Subgroup analysis was performed according to the countries (i.e., developed versus developing countries) where studies were conducted. This suggested that hemoglobin effects in developing countries (*n* = 9 studies) [9–12, 16, 17, 28, 32, 34] were similar to the overall effects with the pooled ORs of 1.69 (95% CI: 1.28–2.23), 1.34 (95% CI: 1.17–1.53), 1.10 (95% CI: 1.02–1.19), and 0.93 (95% CI: 0.80–1.08) for hemoglobin cutoffs <9, <10, <11, and ≥14 g/dL, respectively. However, the hemoglobin effects were lower and nonsignificant in developed countries (*n* = 2 studies) [18, 27] compared with developing countries with the pooled ORs of 1.17 (95% CI: 0.47–2.93) and 1.21 (95% CI: 0.59–2.51) for the cutoffs of <10 and <11 g/dL, respectively. We also pooled data by type of study designs (i.e., cohort versus case-control studies). The result from 9 cohort studies [9–12, 16–18, 28, 34] were also similar to the overall effects with the pooled ORs of 2.91 (95% CI: 1.79–4.74), 1.34 (95% CI: 1.17–1.53), 1.09 (95% CI: 1.01–1.18), and 0.99 (95% CI: 0.86–1.14), respectively.

**Figure 3:**
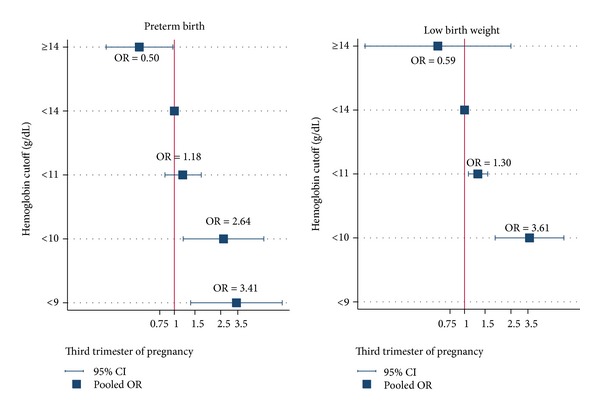
Pooling effects of hemoglobin concentration on pregnancy outcomes in the third trimester.

The increased risk of lower hemoglobin concentration on pregnancy outcomes was also observed in the third trimester following pooling of 7 studies [7, 16, 25–29] with total sample size of 29,879 women. The odds of having PTB was significantly higher in hemoglobin <9 and <10 but not for <11 g/dL when compared with normal hemoglobin with the pooled ORs of 3.41 (95% CI: 1.38–8.42), 2.64 (95% CI: 1.19–5.86), and 1.18 (95% CI: 0.87–1.58), respectively. Conversely, the odds of PTB was 50% reduction (OR =0.50, 95% CI: 0.26–0.97) in hemoglobin ≥14 g/dL; see Table 2 and Figure 3. There was no heterogeneity across studies except for pooling hemoglobin <11 g/dL that was moderately heterogeneous (*I* ^2^ = 45, 95% CI 0–83). There was no evidence of small study effect suggested by the Egger tests with coefficient of 0.168 (*P* = 0.932), −0.445 (*P* = 0.385), and 0.254 (*P* = 0.666) for hemoglobin <9, <10, and <11 g/dL, respectively. The Egger test suggested small study effect in group of hemoglobin ≥14 g/dL with coefficient of 0.113 (*P* = 0.045). A subgroup analysis was performed and suggested that there was no hemoglobin effect in developing countries (*n* = 4 studies) with the pooled OR of 0.95 (95% CI: 0.60–1.50) for hemoglobin <11 g/dL; conversely for developed countries, the odds of PTBs were 3.78 (95% CI: 1.49–9.55), 3.44 (95% CI: 1.54–7.72), 1.66 (95% CI: 0.95–2.92), and 0.53 (95% CI: 0.27–1.03) for hemoglobin cutoffs <9, <10, <11, and ≥14 g/dL compared with hemoglobin 11–13.9 g/dL.

### 3.2. Low Birth Weight

Ten studies [9–12, 17, 27, 28, 31, 32, 34] assessed the association between low hemoglobin concentration in the first trimester and LBW with hemoglobin concentration ranging from 7 to 16 g/dL (Supplementary Table 3). Nine studies [9–12, 17, 28, 31, 32, 34] were conducted in developing countries. The mixed logit model suggested that hemoglobin concentration below 9, 10, and 11 g/dL had, respectively, 2.14 (95% CI: 1.57–2.91), 1.57 (95% CI: 1.30–1.90), and 1.17 (95% CI: 1.03–1.32) times significantly higher risk of LBW, whereas hemoglobin ≥14 g/dL did not increase the risk (OR = 0.89, 95% CI: 0.70–1.13) when compared with pregnant women with hemoglobin concentration 11–13.9 g/dL (Table 2 and Figure 2). The individual ORs were homogeneous across studies for pooling low hemoglobin groups (*I* ^2^ ranged from 0% to 7%) but moderately heterogeneous for hemoglobin ≥ 14 g/dL (*I* ^2^ = 44%).

The Egger test was applied and suggested no evidence of small study effect with coefficients of −0.019 (*P* = 0.322), 0.140 (*P* = 0.513), 0.076 (*P* = 0.725), and 0.251 (*P* = 0.474) for the cutoffs below 9, 10, 11, and above 14 g/dL, respectively. Pooling studies in developing countries and in cohort studies did not change much results (data were not shown).

Six studies [7, 17, 24, 27–29] reported an association between hemoglobin concentration and LBW in the third trimester with hemoglobin cutoff ranged from <10 to <11 g/dL (Table 2). The mixed logistic model was applied and yielded estimated ORs of 3.61 (95% CI: 1.83–7.12), 1.30 (95% CI: 1.08–1.58), and 0.59 (95% CI: 0.14–2.50) for hemoglobin cutoff <10, <11, and ≥14 compared with hemoglobin concentration 11–13.9 g/dL, respectively (Table 2). This suggest that pregnant women with hemoglobin concentration lower than 10 and 11 g/dL in the third trimester were at approximately 3.6 and 1.3 times higher risk, respectively, of having a LBW newborn than pregnant women with hemoglobin concentration of 11–13.9 g/dL. The degrees of heterogeneity were mild (*I* ^2^ = 0%, 95% CI: 0–50) and moderate (*I* ^2^ = 58%, 95% CI: 0–85), respectively, without any evidence of small study effects, with the corresponding coefficients of −0.126 (*P* = 0.226) and −0.58 (*P* = 0.937), respectively. Pooling within 4 studies in developing countries and 9 cohort studies gave similar results (data were not shown). 

### 3.3. Small for Gestational Age

Effects of low hemoglobin concentration in the first trimester on SGA were assessed in 6 studies [9, 12, 17, 18, 27, 28] with the sample size totaling 94, 280 women (Supplementary Table 4). The mixed logistic model suggested that hemoglobin concentrations below 10 and 11 g/dL increased the risk of SGA by 26% (OR: 1.26, 95% CI: 1.09–1.45) and 14% (OR: 1.14, 95% CI: 1.05–1.24), respectively (Table 2 and Figure 2). The hemoglobin effects were mildly heterogeneous for both cutoffs with the *I* ^2^ of 1% and 2%. There was no evidence of small study effects as suggested by the Egger test with coefficients of −0.124 (*P* = 0.402) and 0.229 (*P* = 0.645), respectively. Pooling effects in 4 studies conducted in developing countries did not change much results (data were not shown).

## 4. Discussion

We have performed a systematic review and meta-analysis to assess effects of hemoglobin concentration on pregnancy outcomes according to trimesters. Our results suggest that lower hemoglobin concentration is associated with a higher risk of poor pregnancy outcomes in both first and third trimesters. The risk of PTB, LBW, and small gestational age was approximately 10–17% and 26–57% higher in pregnant women who had a hemoglobin concentration below 10 and 11 g/dL in the first trimester, respectively. In the third trimester, hemoglobin below 11 g/dL increases the risk of LBW by 30% but not for preterm term. Hemoglobin below 10 g/dL in the third trimester also increases the risk of PTB and LBW by 2.6 and 3.6 times, respectively. Hemoglobin ≥14 g/dL did not increase risk in any trimester of pregnancy but conversely reduced a risk of PTB by 50%.

Our results confirm the findings from a previous meta-analysis [19] showing that low hemoglobin concentration in early pregnancy (<20 weeks gestation) was associated with PTB. In addition, we also found that low hemoglobin concentration in the first trimester was a risk of LBW and SGA, which has not been reported in the previous review. These findings may be explained by reduced oxygen transportation from the mother to fetus and may reflect inadequate iron reserves during early pregnancy. Subgroup analysis did not show any clear differences in the effects of low hemoglobin concentration between developing and developed countries. The magnitude of the effect in developing countries was similar to results of other previous studies [35–37]. Pooling studies based on cohorts only did not change the results when compared to pooling all studies with cohorts and case controls.

Our study has a number of strengths. We assessed effects of various hemoglobin cutoffs on pregnancy outcomes stratified by trimesters. The use of hemoglobin cutoff in individual studies varied from 7 to 16 g/dL, and one study had more than one cutoff. We therefore applied a mixed logistic model in order to simultaneously assess hemoglobin effects without inflating type one error. Between-study variations were also taken into account in the mixed logistic model. A degree of heterogeneity was also estimated using multivariate meta-analysis method.

There are, however, some limitations and caveats with our study. Because of the varying cutoffs used in different studies, the only way to reasonably pool data was to expand the summary data to individual level data and then pool based on common thresholds; thus some subjects were excluded if studies used a reference cutoff lower than 11 g/dL. It is also possible that our results are confounded by other factors in which analysis based on summary data could not adjust for. A meta-analysis of individual patient data should be conducted to calibrate hemoglobin cutoff with adjusting for confounders; that is, anemia is a marker of general poor health in developing countries but not in developed countries. Our review also excluded non-English articles due to limitation in translation issue.

## 5. Conclusion

 Our review suggests that hemoglobin below 11 g/dL increases the risk of LBW in both first and third trimesters, PTB and small gestational age in the first trimester. Conversely, hemoglobin 14 g/dL or higher can conversely reduce the risk of PTB in the third trimester, but the result might be affected by publication bias.

## Supplementary Material

Appendix S1 described search strategies that were used for PubMed and SCOPUS search engines.
Table S1 described results of risk of bias assessments for individual included studies.
Table S2 provided summary data for preterm birth in first, second and third trimester of pregnancy according to different levels of hemoglobin concentration for each individual study.
Table S3 provided summary data for low birth weight in first, second and third trimester of pregnancy according to different levels of hemoglobin concentration for each individual study.
Table S4 provided summary data for small for gestational age in first, second and third trimester of pregnancy according to different levels of hemoglobin concentration for each individual study.

## Figures and Tables

**Table 1:** Characteristics of included studies.

Authors, year (Reference number)	Study design	Sample size	Mean age	Country	Trimester	Pregnancy outcomes	Confounding factors
Abeysena et al., 2010 [17]	Prospective cohort	817	26.4 ± 5.5	Sri Lanka	First	PTB, LBW, and SGA	Not specify

Kumar et al., 2010 [34]	Prospective cohort	2,027	24.6 ± 3.82	India	First	LBW	Maternal age, parity, maternal height, maternal weight, BMI, and gestational age

Kidanto et al., 2009 [32]	Case control	1,721	24^a^	Tanzania	First	PTB, LBW, and SB	Not specify

Zhang et al., 2009 [16]	Prospective cohort	160,700	NA	China	First Second Third	PTB	Maternal age at delivery, education, occupation, parity, folic acid, BMI, time of first prenatal visit, and fetal gender

Zhang et al., 2009 [33]	Prospective cohort	164,667	NA	China	First Second Third	SB, ND	Maternal age at delivery, education, occupation, parity, folic acid, BMI, time of first prenatal visit, and fetal gender

Ren et al., 2007 [12]	Retrospective cohort	88,149	25.8 ± 2.9	China	First	PTB, LBW, and SGA	Maternal age, gravidity, education, and BMI

Lee et al., 2006 [29]	Prospective cohort	248	30.6 ± 3.35	Korea	Third	PTB, LBW	Not specify

Mamun et al., 2006 [30]	Retrospective cohort	1,584	22.1 ± 4.3	Bangladesh	Second	SB, PD	Not specify

Monawar Hosain et al., 2006 [31]	Prospective cohort	350	NA	Bangladesh	First	LBW	Not specify

Levy et al., 2005 [11]	Retrospective cohort	153,396	28.3 ± 5.9	Irael	First	PTB, LBW, and PD	Ethnicity, maternal age, placental problems, caesarean delivery, and nonvertex presentation

Little et al., 2005 [15]	Retrospective cohort	222,614	NA	England	First	STB, ND	Prematurity, birth weight

Ronnenberg et al., 2004 [10]	Prospective cohort	405	24.9 ± 1.5	China	First	PTB, LBW	Maternal age, height, BMI, education, exposure to dust, noise, passive smoking, work stress, infant gender, and gestational age

Chang et al., 2003 [26]	Retrospective cohort	918	16.1 ± 1.1	USA	Second Third	PTB, LBW	Parity, BMI, smoking, preeclampsia, and antenatal care

Hämäläinen et al., 2003 [27]	Case control	22,799	28.9 ± 5.2	Finland	First Second Third	PTB, LBW, SGA, and PD	Not specify

Xiong et al., 2003 [28]	Retrospective cohort	16,936	25.0 ± 2.8	China	First	PTB, LBW, and PD	Hospital stay, maternal age, maternal education, parity, gestational age at the first prenatal visit, BMI, hypertensive disorder in pregnancy, vaginal bleeding, and prior spontaneous abortion

Martí et al., 2001 [25]	Case control	543	24.1 ± 6.6	Venezuela	Third	PTB	Placenta abruption, PROM, previous premature labor, ANC visit, and antenatal bleeding

Zhou et al., 1998 [9]	Prospective cohort	829	25.5 ± 3.8	China	First	PTB, LBW, and SGA	Not specify

Onadeko et al., 1996 [24]	Prospective cohort	4,649	NA	Nigeria	Third	LBW, SB	Not specify

Rasmussen and Oian, 1993 [18]	Retrospective cohort	3,074	NA	Norway	First Second	PTB, SGA	Not specify

Knottnerus et al., 1990 [7]	Prospective cohort	796	27^a^	Netherland	Third	PTB, LBW	Pregnancy induced hypertension

^a^Median. LBW: low birth weight; NA: not available; ND: neonatal deaths; PD: perinatal deaths; PTB: preterm birth; SGA: small for gestational age; SB: still births.

**Table 2:** Association between hemoglobin concentration and pregnancy outcomes.

Outcome	Trimester	Number of studies	Number of subjects	Hemoglobin cutoff	Pooled OR	95% CI	*I* ^2^	95% CI
Preterm birth	First			<9	1.72	1.30–2.26	4	0–45
		11	192,870	<10	1.33	1.17–1.52	10	0–59
				<11	1.10	1.02–1.19	5	0–44
	Reference			11–13.9	1.00			
				≥14	0.99	0.86–1.14	31	0–74
							
	Third			<9	3.41	1.38–8.42	0	0–6
		7	29,879	<10	2.64	1.19–5.86	0	0–45
				<11	1.18	0.83–1.70	45	0–83
	Reference			11–13.9	1.00			
				≥14	0.50	0.26–0.97	0	0–48

Low birth weight	First			<9	2.14	1.57–2.91	0	0–11
		10	106,892	<10	1.57	1.30–1.90	7	0–43
				<11	1.17	1.03–1.32	2	0–38
	Reference			11–13.9	1.00			
				≥14	0.89	0.70–1.13	44	0–84
							
	Third	6	9,285	<10	3.61	1.83–7.12	0	0–50
				<11	1.30	1.08–1.58	58	0–91
	Reference			11–13.9	1.00			
				≥14	0.59	0.14–2.50	0	0

Small for gestational age	First			<9	1.37	0.73–2.56	7	0–60
		6	94,280	<10	1.26	1.09–1.45	1	0–47
				<11	1.14	1.05–1.24	2	0–55
	Reference			11–13.9	1.00			
				≥14	0.97	0.84–1.12	20	0–68
