# Supplementary material for: Hemoglobin Concentration and Pregnancy Outcomes: A Systematic Review and Meta-Analysis
Source: Biomed Res Int. 2013 Jul 25;2013:769057. doi: 10.1155/2013/769057 (PMC3741929; doi:10.1155/2013/769057)
Supplement: Supplementary file 1 — Appendix S1 described search strategies that were used for PubMed and SCOPUS search engines. Table S1 described results of risk of bias assessments for individual included studies. Table S2 provided summary data for preterm birth in first, second and third trimester of pregnancy according to different levels of hemoglobin concentration for each individual study. Table S3 provided summary data for low birth weight in first, second and third trimester of pregnancy according to different levels of hemoglobin concentration for each individual study. Table S4 provided summary data for small for gestational age in first, second and third trimester of pregnancy according to different levels of hemoglobin concentration for each individual study. [file 769057.f1.pdf]

Appendix S1 : Search strategies for Medline and Scopus
P 1. pregnancy
2. pregnant women
3. 1 or 2
I 4. hemoglobin
5. haemoglobin
6. anemia
7. anaemia
8. hematologic parameter
9. haematologic parameter
10. 4 or 5 or 6 or 7 or 8 or 9
11. 3 AND 10
O 12. mortality
13. preterm birth
14. preterm delivery
15. low birth weight
16. small for gestational age
17. 12 or 13 or 14 or 15 or 16
18 11 AND 17

Table S1. Risk of bias assessments for individual included studies

**Author(year)**	**Representative of studied subjects**	**Ascertainment of outcomes**	**Ascertainment of hemoglobin measurement**	**Confounding bias**
Abeysena et al., (2010)[17]	No	Yes	No	Yes
Kumar et al., (2010)[34]	Yes	Yes	Yes	Yes
Kidanto et al., (2009)[32]	Yes	Yes	Yes	Yes
Zhang et al., (2009)[16]	Yes	Yes	Yes	Yes
Zhang et al., (2009)[33]	Yes	Yes	Yes	No
Ren et al., (2007)[12]	Yes	Yes	Yes	Yes
Lee et al., (2006)[29]	Yes	Yes	Yes	Yes
Mamun et al., (2006)[30]	Yes	Yes	Yes	Yes
Hosain(2006)[31]	Yes	Yes	Yes	Yes
Levy et al., (2005)[11]	Yes	Yes	Yes	Yes

Little et al., (2005)[15]	Yes	Yes	Yes	Yes
Ronnenberg et al., (2004)[10]	Yes	Yes	Yes	No
Chang et al., (2003)[26]	Yes	Yes	No	Yes
Hamalainen et al., (2003)[27]	Yes	Yes	No	Yes
Xiong et al., (2003)[28]	Yes	Yes	No	Yes
Marti(2001)[25]	Yes	Yes	Yes	No
Zhou et al., (1998)[9]	Yes	Yes	No	Yes
Onadenko(1996)[24]	Yes	Yes	Yes	Yes
Rasmussen and Oian, (1993)[18]	Yes	Yes	Yes	Yes
Knottnerus et al., (1990)[7]	Yes	Yes	No	Yes

Each item is asked whether there is low risk of bias? Yes=low risk, No = high risk

Table S2. Frequency data about hemoglobin concentration and preterm birth

**Trimester**	**Author, Year [reference no.]**	**Hb cutoff(g/dl)**	**event**	**no-event**	**OR (95% CI)**	**Adjusted OR (95% CI)**
1	Abeysena et al., 2010[17]	< 10.4	2	18	0.84(0.19-3.67)	0.64(0.08-5.15)
		10.4-13.9 (reference)	75	564	1.0	1.0
		> 13.9	3	32	0.70(0.21-2.36)	0.37(0.05-2.80)
	Kumar et al., 2010[34]	< 7	6	8	2.47(0.70-8.16)	NA
		≥ 7 (reference)	484	1,549	1.0	NA
	Kidanto et al., 2009[32]	< 7	37	63	4.1(2.5-6.6)	NA
		7.0-8.9	67	336	1.4(0.96-2.0)	NA
		9.0-10.9	112	559	1.4(1.01-1.9)	NA
		≥ 11 (reference)	68	479	1.0	NA
	Zhang et al., 2009[16]	< 10	480	10,210	1.11(1.00-1.22)	NA
		≥ 10 (reference)	3,545	83,377	1.0	NA

	Ren et al., 2007[12]	< 8.0	10	100	2.17(1.00-4.17)	1.86(0.90-3.84)
		8.0-9.9	218	3,610	1.31(1.13-1.52)	1.34(1.16-1.55)
		10.0-11.9 (reference)	1,937	42,091	1.0	1.0
		12.0-13.9	1,570	34,117	1.00(0.93-1.07)	0.98(0.91-1.04)
		14.0-15.9	176	4,123	0.93(0.79-1.09)	0.91(0.77-1.06)
		≥ 16	6	193	0.68(0.24-1.50)	0.42(0.14-1.33)
	Levy et al., 2005[11]	< 10	1,411	11,793	1.20(1.13-1.27)	1.2(1.1-1.2)
		≥ 10 (reference)	12,683	127,509	1.0	1.0
	Ronnenberg et al., 2004[10]	< 9.5	6	34	2.09(0.54-7.83)	2.9(0.8-11.3)
		9.5-11.9	14	261	0.63(0.23-1.93)	0.7(0.3-1.9)
		≥ 12 (reference)	7	83	1.0	1.0
	Hamalainen et al., 2003[27]	< 10	5	64	1.80(0.57-4.45)	1.80(0.72-4.49)
		≥ 10 (reference)	920	21,282	1.0	1.0
	Xiong et al., 2003[28]	< 10	59	1,428	1.07(0.81-1.41)	1.01(0.75-1.37)
		≥ 10 (reference)	482	12,541	1.0	1.0
	Zhou et al., 1998[9]	< 9.0	5	27	3.73(1.36-10.23)	NA
		9.0-9.9	12	97	2.63(1.17-5.90)	NA
		10.0-10.9	18	245	1.64(0.77-3.47)	NA
		11.0-11.9 (reference)	10	229	1.0	NA
		12.0-12.9	6	123	1.11(0.41-2.99)	NA
	Rasmussen and Oian, 1993[18]	< 11	8	99	1.26(0.52-2.64)	NA
		11-14 (reference)	158	2,464	1.0	NA
		≥ 14	33	312	1.65(1.08-2.46)	NA
2	Zhang et al., 2009[16]	< 10	772	18,783	0.95(0.87-1.02)	NA
		≥ 10 (reference)	3,253	74,804	1.0	NA
	Chang et al., 2003[26]	≤ 10.5	5	59	0.98(0.26-3.09)	0.98(0.37-2.64)
		10.6-12.0	13	150	1.0	1.0

		(reference)				
		> 12.0	11	52	2.44(0.92-6.29)	2.19(1.04-4.61)
	Hamalainen et al., 2003[27]	< 10	11	261	0.97(0.48-1.78)	0.97(0.53-1.48)
		≥ 10 (reference)	920	21,282	1.0	1.0
3	Zhang et al., 2009[16]	< 10	871	23,716	0.81(0.75-0.88)	NA
		≥ 10 (reference)	3,154	69,871	1.0	NA
	Lee et al., 2006[29]	< 10.8	11	44	2.93(0.79-13.47)	NA
		10.8-11.9 (reference)	4	47	1.0	NA
		≥ 12	3	60	0.59(0.08-3.67)	NA
	Chang et al., 2003[26]	≤ 10.5	24	266	0.73(0.41-1.26)	0.76(0.48-1.21)
		10.6-12.0 (reference)	44	356	1.0	1.0
		> 12.0	12	67	1.45(0.66-2.97)	1.43(0.80-2.57)
	Hamalainen et al., 2003[27]	< 10	14	242	1.33(0.72-2.30)	1.53(0.88-2.64)
		≥ 10 (reference)	920	21,282	1.0	1.0
	Xiong et al., 2003[28]	< 10	81	2,920	0.65(0.51-0.82)	0.72(0.56-0.92)
		≥ 10 (reference)	531	12,541	1.0	1.0
	Marti et al., 2001[25]	5-8	13	7	3.71(1.27-11.66)	3.92(1.58-9.72)
		9-9.99	16	10	3.20(1.25-8.47)	3.41(1.54-7.54)
		10-10.99	25	34	1.47(0.75-2.86)	1.54(0.89-2.66)
		11-11.99	42	77	1.09(0.63-1.88)	1.11(0.73-1.71)
		12-12.99 (reference)	48	96	1.0	1.0
		13-13.99	25	87	0.57(0.31-1.04)	0.50(0.31-0.82)
		> 14	12	51	0.47(0.21-1.00)	0.43(0.22-0.82)
	Knottnerus et al., 1990[7]	≤ 6.9	8	201	0.40(0.13-1.22)	NA
		7.0-7.9	17	470	0.37(0.15-0.96)	NA
		≥ 8.0 (reference)	9	91	1.0	NA

Table S3. Frequency data for hemoglobin groups and low birth weight

Trimester	Author, Year [reference no.]	Hb cutoff(g/dl)	event	no-event	OR (95% CI)	Adjusted OR (95% CI)
1	Abeysena et al., 2010[17]	< 10.4	3	17	1.43(0.26-5.14)	1.43(0.41-5.00)
		10.4-13.9 (reference)	70	569	1.0	1.0
		> 13.9	9	26	2.81(1.11-6.49)	2.81(1.27-6.25)
	Kumar et al., 2010[34]	< 10	217	644	1.02(0.83-1.25)	NA
		≥ 10 (reference)	289	877	1.0	NA
	Kidanto et al., 2009[32]	< 7	30	70	3.8(2.3-6.3)	NA
		7.0-8.9	72	331	1.7(1.2-2.6)	NA
		9.0-10.9	86	585	1.2(0.85-1.7)	NA
		≥ 11 (reference)	59	488	1.0	NA
	Ren et al., 2007[12]	< 8.0	5	105	2.60(0.82-6.28)	2.16(0.79-5.89)
		8.0-9.9	103	3,725	1.51(1.21-1.86)	1.44(1.17-1.78)
		10.0-11.9 (reference)	793	43,235	1.0	1.0
		12.0-13.9	678	35,009	1.06(0.95-1.17)	1.08(0.97-1.20)
		14.0-15.9	64	4,235	0.82(0.63-1.07)	0.90(0.70-1.17)
		≥ 16	2	195	0.56(0.07-2.05)	0.78(0.19-3.15)
	Hosain et al., 2006[31]	< 8	51	116	2.00(1.18-3.40)	NA
		≥ 8	33	150	1.0	NA

		(reference)				
	Levy et al, 2005[11]	< 10	1,386	11,818	1.13(1.07-1.20)	1.1(1.1-1.2)
		≥ 10 (reference)	13,172	127,020	1.0	1.0
	Ronnenberg et al, 2004[10]	< 9.5	7	33	3.61(0.90-15.32)	6.5(1.6-26.7)
		9.5-11.9	21	254	1.41(0.49-4.92)	2.0(0.7-5.9)
		≥ 12 (reference)	5	85	1.0	1.0
	Hamalainen et al., 2003[27]	< 10	6	63	3.19(1.13-7.38)	3.14(1.35-7.28)
		≥ 10 (reference)	643	21,559	1.0	1.0
	Xiong et al., 2003[28]	< 10	39	1,448	0.83(0.59-1.16)	0.75(0.53-1.07)
		≥ 10 (reference)	404	12,619	1.0	1.0
	Zhou et al., 1998[9]	< 9.0	2	30	2.99(0.60-14.76)	NA
		9.0-9.9	8	101	3.27(1.09-9.77)	NA
		10.0-10.9	15	248	2.73(1.01-7.39)	NA
		11.0-11.9 (reference)	5	234	1.0	NA
		12.0-12.9	5	124	1.85(0.55-6.28)	NA
		≥ 13.0	3	54	2.52(0.62-6.28)	NA
2	Chang et al., 2003[26]	≤ 10.5	6	58	1.57(0.45-5.02)	1.52(0.58-4.03)
		10.6-12.0 (reference)	10	152	1.0	1.0
		> 12.0	12	51	3.58(1.32-9.79)	3.09(1.40-6.82)
	Hamalainen et al., 2003[27]	< 10	5	267	0.63(0.20-1.49)	0.61(0.25-1.48)
		≥ 10 (reference)	643	21,559	1.0	1.0
3	Lee et al., 2006[29]	< 10.8	11	43	3.01(0.80-13.79)	NA
		10.8-11.9 (reference)	4	47	1.0	NA
		≥ 12	3	61	0.58(0.08-3.61)	NA
	Chang et al., 2003[26]	≤ 10.5	21	268	0.68(0.37-1.22)	0.69(0.42-1.14)
		10.6-12.0 (reference)	41	358	1.0	1.0

	> 12.0	15	63	2.08(1.01-4.11)	1.88(1.11-3.19)
Hamalainen et al., 2003[27]	< 10	8	248	1.08(0.46-2.18)	1.28(0.63-2.62)
	≥ 10 (reference)	643	21,559	1.0	1.0
Xiong et al., 2003[28]	< 10	81	2,920	0.86(0.67-1.09)	0.90(0.71-1.61)
	≥ 10 (reference)	401	12,541	1.0	1.0
Onadenko et al., 1996[24]	< 10	16	28	4.39(2.18-8.52)	NA
	10.0-10.9	146	857	1.31(1.04-1.63)	NA
	11.0-11.9 (reference)	260	1,997	1.0	NA
	12.0-12.9	100	873	0.88(0.68-1.13)	NA
	13.0-13.9	18	215	0.64(0.37-1.06)	NA
	14.0-14.9	2	27	0.57(0.07-2.29)	NA
Knottnerus et al., 1990[7]	≤ 6.9	10	199	0.28(0.11-0.71)	NA
	7.0-7.9	19	468	0.23(0.11-0.51)	NA
	≥ 8.0 (reference)	15	85	1.0	NA

Table S4. Frequency data for hemoglobin groups and small for gestational age

Trimester	Author, Year [reference no.]	Hb cutoff(g/dl)	event	no-event	OR (95% CI)	Adjusted OR (95% CI)
1	Abeysena et al., 2010[17]	< 10.4	2	13	0.82(0.09-3.70)	0.81(0.18-3.67)
		10.4-13.9 (reference)	96	509	1.0	1.0
		> 13.9	7	24	1.55(0.55-3.83)	1.55(0.65-3.70)
	Ren et al., 2007[12]	< 8.0	8	103	1.58(0.66-3.23)	1.49(0.69-3.23)
		8.0-9.9	211	3,617	1.35(1.16-1.57)	1.13(0.98-1.31)
		10.0-11.9 (reference)	2,069	41,959	1.0	1.0
		12.0-13.9	1,535	34,152	0.91(0.85-9.76)	0.98(0.91-1.05)
		14.0-15.9	181	4,118	0.89(0.76-1.04)	0.99(0.84-1.16)
		≥ 16	6	191	0.64(0.23-1.41)	0.61(0.22-1.65)
	Xiong et al., 2003[28]	< 10	80	1,407	0.81(0.64-1.02)	0.78(0.61-1.01)
		≥ 10 (reference)	860	12,163	1.0	1.0
	Hamalainen et al, 2003[27]	< 10	6	63	0.94(0.33-2.16)	0.94(0.41-2.17)
		≥ 10 (reference)	2,051	20,151	1.0	1.0
	Zhou et al., 1998[9]	< 9.0	3	29	0.80(0.26-2.48)	NA
		9.0-9.9	13	96	1.02(0.55-1.89)	NA
		10.0-10.9	31	232	1.01(0.62-1.63)	NA
		11.0-11.9 (reference)	28	211	1.0	NA
		12.0-12.9	15	114	0.99(0.55-1.79)	NA
		≥ 13.0	5	52	0.75(0.30-1.85)	NA
	Rasmussen and Oian, 1993[18]	< 11	5	102	2.81(0.85-7.26)	NA
		11-14 (reference)	45	2,577	1.0	NA
		≥ 14	10	335	1.61(0.72-3.28)	NA
2	Hamalainen et al.,	< 10	21	251	0.82(0.50-1.29)	0.80(0.51-1.25)

	2003[27]	> 10 (reference)	2,051	20,151	1.0	1.0
	Rasmussen and Oian, 1993[18]	< 10.0	4	219	0.95(0.25-2.63)	NA
		10.0-13.0 (reference)	47	2,441	1.0	NA
		> 13.0	9	154	3.04(1.28-6.41)	NA
3	Hamalainen et al., 2003[27]	< 10	18	238	0.74(0.43-1.20)	0.75(0.46-1.21)
		> 10 (reference)	2,051	20,151	1.0	1.0
	Xiong et al., 2003[28]	< 10	183	2,818	0.93(0.79-1.10)	0.93(0.79-1.10)
		> 10 (reference)	841	12,101	1.0	1.0
